# Study of Inflammatory Biomarkers in Treatment-Naive HIV Patients and Their Correlation With Clusters of Differentiation 4 (CD4) Count

**DOI:** 10.7759/cureus.66234

**Published:** 2024-08-05

**Authors:** Shafat I Bhati, Ahmad Alam, Mohammad Owais, Anjum Parvez, Haroon S Khan, Raihan Mannan

**Affiliations:** 1 General Medicine, Jawaharlal Nehru Medical College and Hospital, Aligarh Muslim University, Aligarh, IND; 2 Rajiv Gandhi Centre for Diabetes and Endocrinology, Jawaharlal Nehru Medical College and Hospital, Aligarh Muslim University, Aligarh, IND; 3 Interdisciplinary Biotechnology, Aligarh Muslim University, Aligarh, IND; 4 Physiology, All India Institute of Medical Sciences, Patna, Patna, IND

**Keywords:** interleukins, hiv, cd4, biomarkers, aids

## Abstract

Introduction

The human immunodeficiency virus (HIV) primarily targets clusters of differentiation 4 (CD4)+ T cells and other immune cells, leading to immune dysfunction. Cytokines such as interleukin (IL)-23 and IL-27 have complex roles in HIV-associated disease progression, affecting viral replication and immune responses. This study aimed to explore the correlation between HIV-related CD4 lymphopenia and the inflammatory cytokines IL-23 and IL-27 in treatment-naive HIV patients.

Materials and methods

This is a single-center, prospective, observational study conducted at the Antiretroviral Treatment (ART) Center of Jawaharlal Nehru Medical College and Hospital, Aligarh Muslim University, Aligarh, Uttar Pradesh, India. Sixty-five treatment-naive HIV seropositive patients were recruited in this study. Quantitative estimation of inflammatory biomarkers (IL-23 and IL-27) was performed using enzyme-linked immunosorbent assay (ELISA). The fluorescent-activated cell sorter count (FACSCount) technology was used to determine the CD4+ T-cell count.

Results

Our study revealed that HIV-infected individuals had significantly higher levels of IL-23 (868.9±246.7 pg/mL vs 98.3±86.6 pg/mL, p < 0.01) and IL-27 (1629.5±518.5 pg/mL vs 291.3±225.2 pg/mL, p < 0.01) compared to healthy controls. Additionally, we found a strong positive correlation between CD4 count and IL-23 titers (r = 0.93, p < 0.01), as well as between CD4 count and IL-27 titers (r = 0.92, p < 0.01) in HIV-positive individuals.

Conclusion

The findings suggest that these cytokines respond to HIV infection and may potentially play a crucial role in restraining HIV replication and slowing down the progression of the disease.

## Introduction

In 1981, the emergence of acquired immune deficiency syndrome (AIDS) marked a watershed moment in medical history, initially impacting young homosexual men and posing perplexing questions for the scientific community [1]. This pursuit led to the identification of human immunodeficiency virus type 1 (HIV-1) as the causative agent, ushering in one of the most devastating infectious diseases in history [2]. Its most profound impact has been in developing nations, particularly sub-Saharan Africa, where young adults bear a disproportionate burden of infection. As of 2022, an estimated 39 million individuals were living with HIV/AIDS, with approximately 0.63 million deaths attributed to AIDS-related illnesses worldwide. In India, approximately 2.5 million individuals were living with HIV, with around 40 thousand deaths due to AIDS-related diseases [3].

Significant advancements in treating HIV largely emerged with the introduction of highly active antiretroviral therapy (HAART), which effectively reduces viral loads in the peripheral blood to undetectable levels. However, despite the availability of HAART, the virus persists within reservoirs in the body, primarily within the genomes of non-activated T lymphocytes and other latent cells [4]. Therefore, there is a pressing need to explore immunology-based therapies that empower the host to maintain control over the infection. In this context, cytokines emerge as promising targets for immunomodulatory interventions.

HIV infection is associated with a progressive decline in clusters of differentiation 4 (CD4) cell counts, sustained immune activation, and altered cytokine levels in the blood. Dysregulation of cytokine signaling is hypothesized to contribute to immune deficiency associated with HIV. Although the T helper (Th) switch doesn't entirely elucidate how HIV spreads, it's notable that early-stage HIV infection is marked by a Th1-predominant profile with increased production of cytokines like IL-2 and interferon (IFN). In contrast, late-stage HIV infection is characterized by a Th2-predominant profile with heightened levels of cytokines such as IL-4 and IL-10. Recent research has uncovered a growing array of potential players in the pathogenesis of HIV, including cytokines like IL-7, IL-15, and IL-21, as well as IL-17, IL-18, IL-19, IL-20, IL-23, and IL-27 [5]. Among these, two cytokines from the IL-12 family, namely IL-23 and IL-27, hold particular significance. IL-23 is associated with the activation of the Th17 pathway [6], while IL-27 is known for promoting Th1 responses and enhancing the survival of naïve T and B cells [7].

The primary focus of our study is to investigate the impact of HIV infection on the levels of IL-23 and IL-27 among treatment-naive individuals. Understanding how HIV affects these cytokines offers valuable insights into the underlying mechanisms of disease and potential therapeutic targets.

## Materials and methods

This was a prospective, observational study conducted at the Department of Medicine, Jawaharlal Nehru Medical College and Hospital (JNMCH) and the Interdisciplinary Biotechnology Unit, Aligarh Muslim University (AMU), Aligarh, India, from January 2016 to January 2018. Patients were recruited from the Antiretroviral Treatment (ART) Centre, JNMCH, AMU. The study was approved by the Institutional Ethics Committee of JNMCH, AMU (approval number: 231IEC, JNMCH) and conducted in accordance with the Declaration of Helsinki. Informed consent was obtained from all participants.

Study participants

Patients who were 18 years or older, newly diagnosed with HIV, and gave consent were included in the study. Hospitalized patients, those with acute illness in the last two weeks, concomitant viral hepatitis B or hepatitis C, a history of cardiovascular disease, or patients already on HAART were excluded.

**Figure 1:**
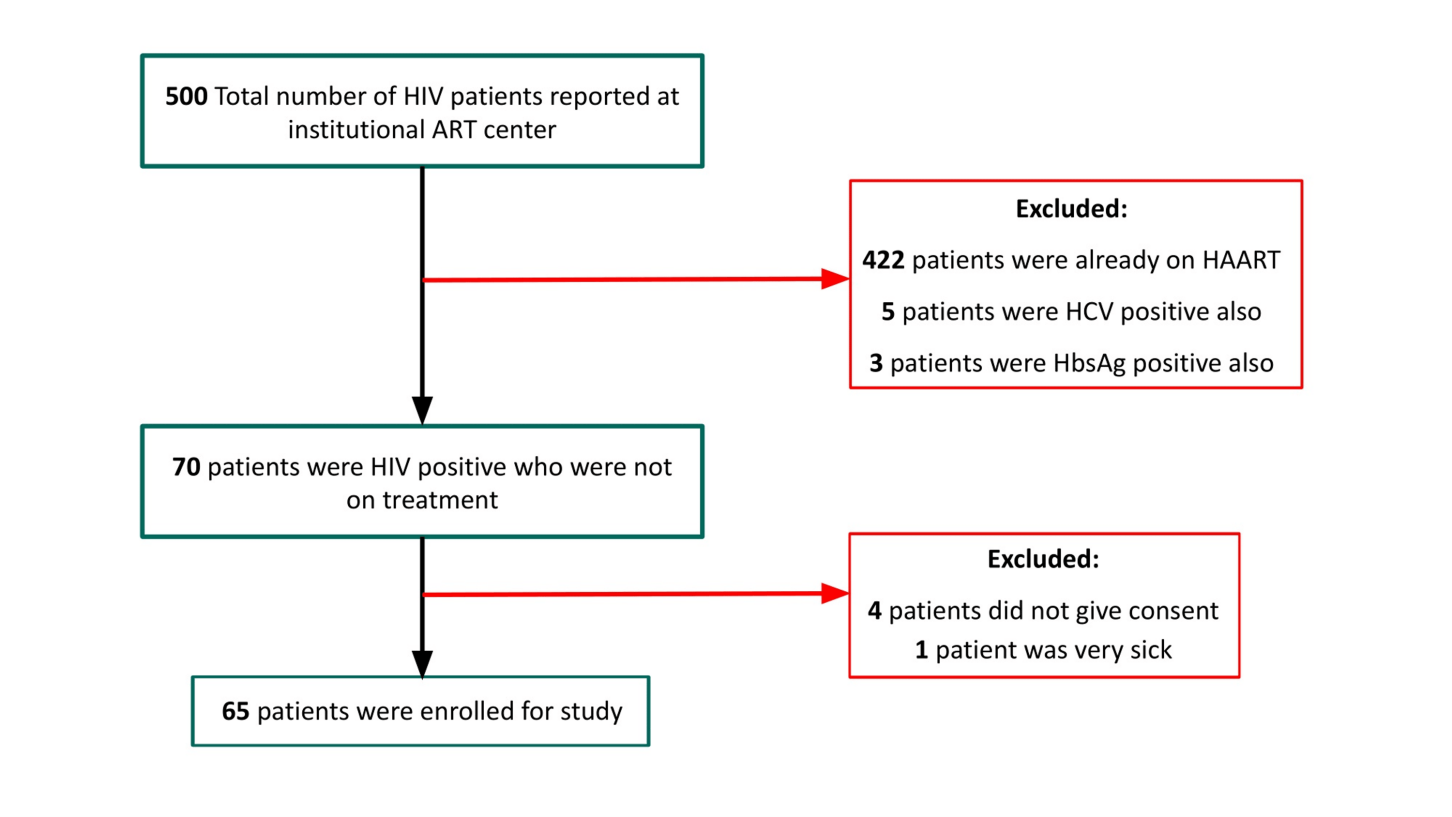
Flowchart depicting patient enrollment in the study. HIV: human immunodeficiency virus; HAART: highly active antiretroviral therapy; ART: antiretroviral therapy; HCV: hepatitis C virus; HBsAg: hepatitis B surface antigen

A total of 65 patients were included in the study (Figure 1). Convenience sampling was used, considering that our study is hospital-based. The control group comprised 10 healthy blood donors. They underwent a thorough examination and blood screening for infections before enrollment. Those with negative screening for hepatitis B, hepatitis C, malaria, syphilis, and HIV were enrolled as controls.

Data collection

Enrolled patients were interviewed in detail regarding sociodemographic information such as age, gender, residence, education, sexual orientation, marital status, and mode of HIV transmission. Patients were given information about the known routes of HIV transmission and asked to select the most likely route for HIV acquisition in their case to record the mode of transmission of HIV. If a patient was unsure of their possible route of HIV acquisition or refused to disclose sexual exposure history, declined to disclose prior intravenous (IV) drug use, or refused to disclose or could not recall past medical, surgical, or blood transfusion history, that patient was labeled as "unknown" in the possible mode of transmission (MOT). The WHO standards for clinical staging of HIV/AIDS in adults were used to establish the clinical stage [8].

Measurement of plasma cytokines

Blood samples were collected from each patient in anticoagulant tubes containing ethylenediaminetetraacetic acid (EDTA). The blood samples were centrifuged for 15 minutes at 2000 rpm to separate plasma, following which plasma samples were immediately stored at -80º C until use. Plasma levels of IL-23 and IL-27 were measured by the enzyme-linked immunoassay (ELISA) method using a commercial kit (Diaclone Research SAS, Besançon, France) by employing appropriate and specific biotinylated antibody pairs according to the manufacturer’s protocol.

The sensitivity of kits for IL-23 and IL-27 were 20 pg/ml and 12.8 pg/ml, respectively. Briefly, 100 μl of the purified capture antibodies were adsorbed overnight on polystyrene microtitre plates at 4⁰C in the carbonate-bicarbonate buffer of pH 9.5. Plates were washed five times with phosphate-buffered saline with Tween 20 (PBST) and blocked with 5% skimmed milk. After the usual washing steps, 100 μl of the patient’s plasma was dispensed in each well to determine its cytokine content. After the stipulated incubation time, the plates were thoroughly washed and incubated with a biotinylated mouse anti-human cytokine detection antibody. Afterward, the plate was washed three times with PBST. Subsequently, 100 μl of the streptavidin-horseradish peroxidase (HRP) conjugate was added to each well, and the plate was incubated for 30 minutes at room temperature. The plate was again washed three times with PBST, and finally, a colored complex was developed with tetramethylbenzidine (TMB). The reaction was then stopped with 2N H_2_SO_4_, and the absorbance was read at 450 nm with a microtitre ELISA plate reader (Bio-Rad Laboratories, Inc., Hercules, California, United States) within 15 minutes after stopping the reaction. A known specific recombinant cytokine was used as a standard for calculating the level of the given cytokine in the samples tested, and the concentration was expressed as pg/mL relative to the concentration of the standard as determined by plotting the standard curve.

Estimation of CD4 count

The anticoagulated whole blood sample was incubated with fluorescent antibodies. The processed sample was then analyzed, and the CD4+ T-cell count was determined by flow cytometry using the FACSCount system (Becton, Dickinson and Company, Franklin Lakes, New Jersey, United States) as per the manufacturer’s instructions.

Statistical analysis

Continuous quantitative variables were presented as mean ± standard deviation, while qualitative variables were expressed as percentages. Independent samples t-test was used to compare plasma cytokines between patients with HIV and healthy controls. Pearson’s correlation coefficient was used to evaluate the association between plasma cytokine titers and CD4 cell counts. Statistical analyses were performed using the IBM SPSS Statistics for Windows, Version 25.0 (Released 2017; IBM Corp., Armonk, New York, United States). A p-value of <0.05 was considered statistically significant.

## Results

In this prospective, observational study, 65 HIV seropositive treatment-naive patients were enrolled. The sociodemographic profiles of the patients are shown in Table 1.

**Table 1:** Sociodemographic profile of the HIV patients Age is expressed as mean±SD, and qualitative variables are expressed as frequency (percentages).

Characteristics	Variables	Frequency (Percentage)
Age (years), mean ± SD		33±7.7
Age groups (years)	18-24	6 (9.2)
	25-44	50 (76.9)
	45-49	6 (9.2)
	>50	3 (4.6)
Gender	Male	14 (75.4)
	Female	6 (24.6)
	Transgender	0
Mode of transmission	Heterosexual	53 (81.5)
	Blood transfusion	5 (7.7)
	Injectible drug abuse	3 (4.6)
	Unknown	2 (3.1)
	Homosexual	1 (1.5)
	Mother-to-child transmission	1 (1.5)
Marital status	Single/Unmarried	10 (12.3)
	Married	48 (73.8)
	Divorced/Separated	5 (7.7)
	Spouse expired	4 (6.1)
Level of education	Illiterate	20 (30.8)
	Primary school	14 (21.5)
	Secondary school	22 (33.8)
	Graduate	9 (13.8)
WHO clinical staging	Stage 1	10 (15.4)
	Stage 2	27 (41.5)
	Stage 3	22 (33.8)
	Stage 4	6 (9.2)

**Figure 2:**
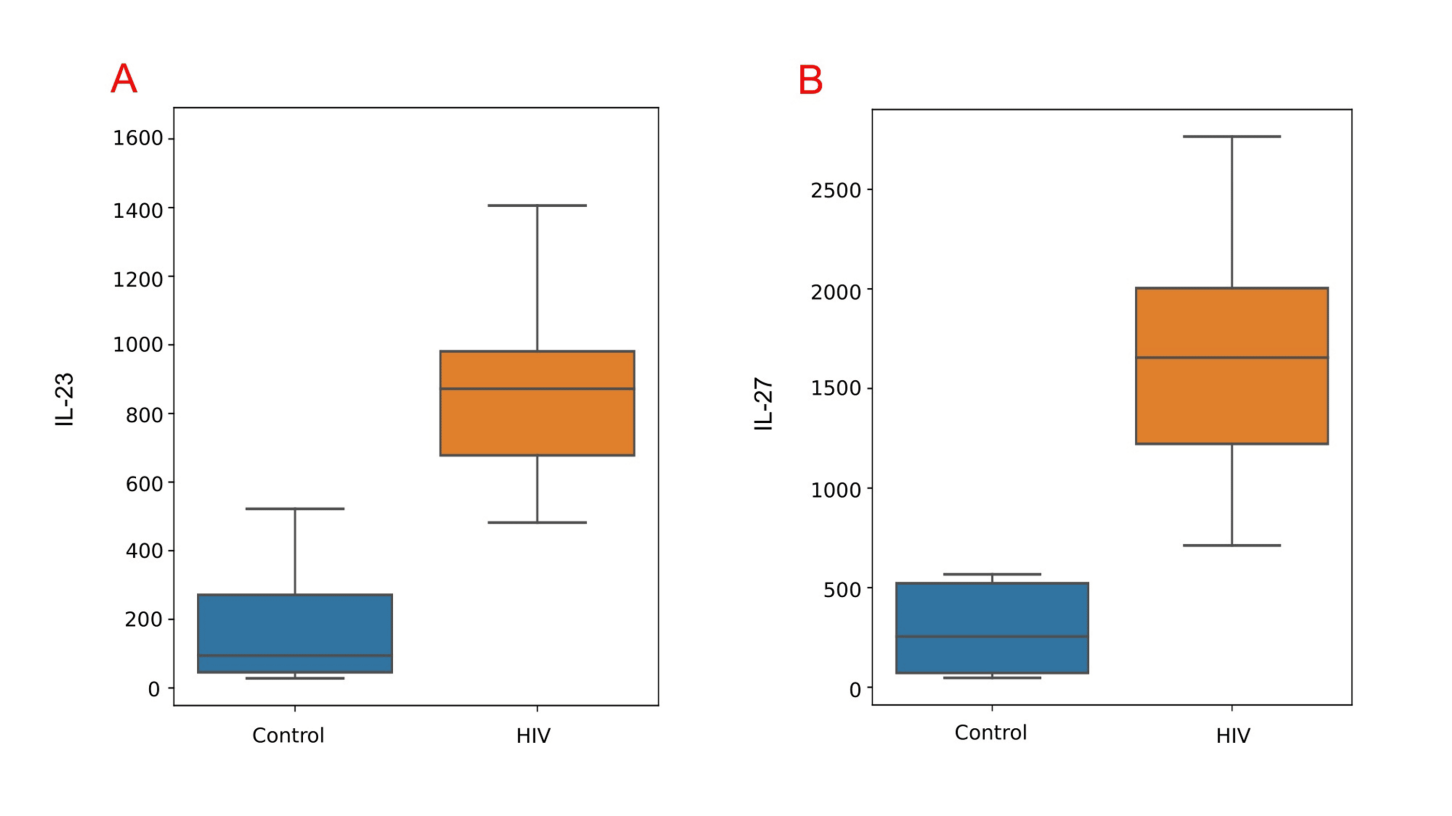
Box plots of IL-23 (A) and IL-27 (B) in controls and treatment-naive HIV patients IL: interleukin

Comparison of CD4 counts, IL-23, and IL-27 levels between the control group (n=10) and the patient group (n=65) revealed significant differences. In healthy individuals, CD4 counts were substantially higher than in HIV-positive individuals (960.7±292.6 cells/μL vs. 375.6±197.4 cells/μL, p < 0.001). Furthermore, HIV-infected individuals exhibited significant upregulation of IL-23 (868.9±246.7 pg/mL vs. 98.3±86.6 pg/mL, p < 0.01) and IL-27 (1629.5±518.5 pg/mL vs. 291.3±225.2 pg/mL, p < 0.01) compared to healthy controls (Table 2). Box plots illustrated the distribution of IL-23 and IL-27 in both groups, highlighting the observed differences (Figure 2).

**Table 2:** CD4 count and cytokine levels in controls and treatment-naive HIV patients Data are presented as mean ± SD. An independent samples t-test was used for statistical analysis, with a p-value of <0.05 considered significant. CD4: clusters of differentiation 4

Characteristics	Control (n=10), mean ± SD	HIV (n=65), mean ± SD	p-value
Age (years)	28±5.9	33±7.7	
CD4 count (cells/µL)	960.7±292.6	375.6±197.4	<0.01
IL-23 (pg/ml)	98.3±86.6	868.9±246.7	<0.01
IL-27 (pg/ml)	291.3±225.2	1629.5±518.5	<0.01

Association between cytokines and CD4+ count

**Figure 3:**
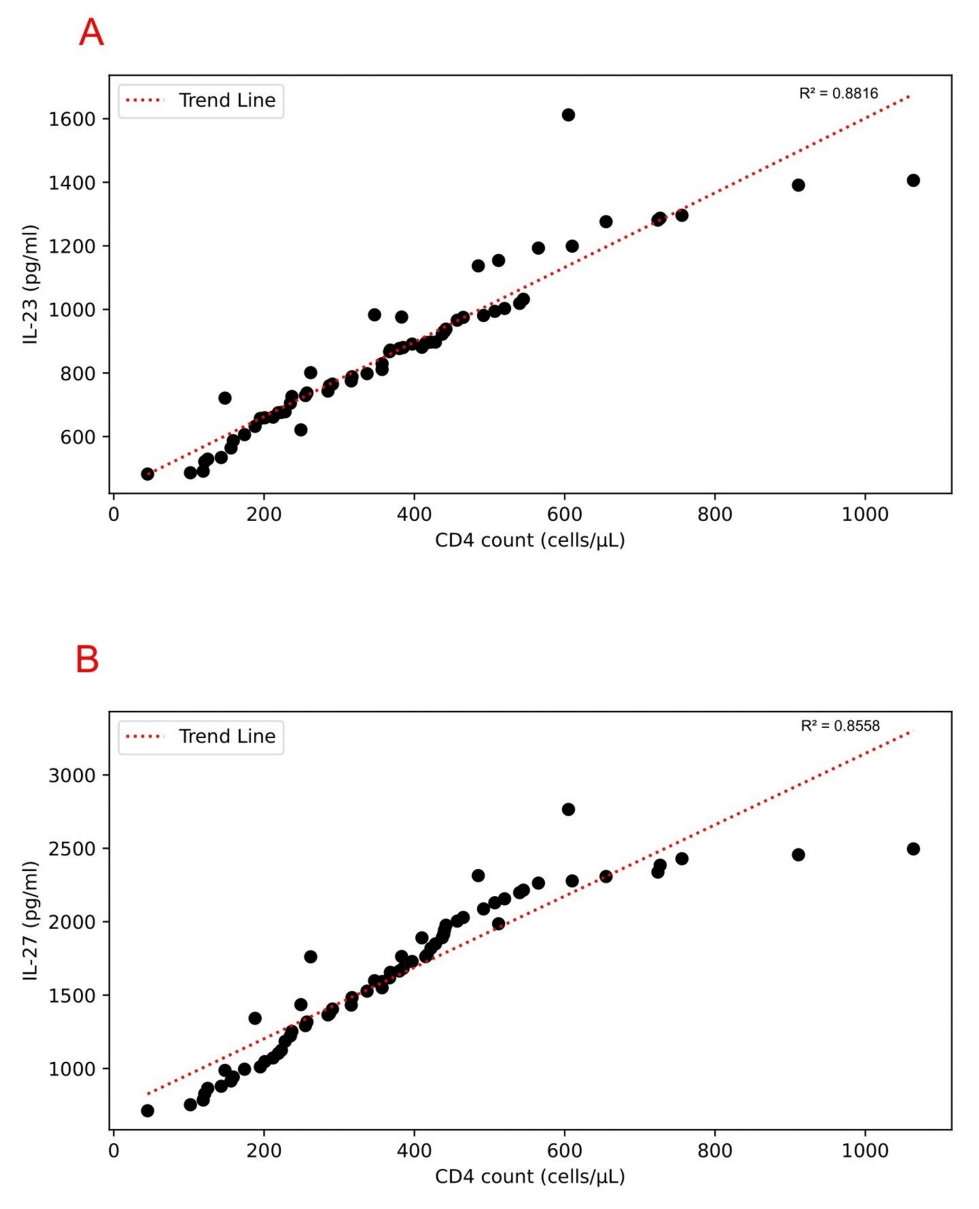
Correlation between plasma cytokine titers ((A) IL-23; (B) IL-27) and CD4 count in treatment-naive HIV patients IL: interleukin

Additionally, within the patient group, there was a strong positive correlation between CD4 count and IL-23 titers (r = 0.93, p < 0.01), as well as between CD4 counts and IL-27 titers (r = 0.92, p < 0.01) in treatment-naive HIV patients (Figure 3).

## Discussion

This prospective, observational study examined the correlation between HIV-related CD4 lymphopenia and inflammatory cytokines, namely IL-23 and IL-27. In our study, 65 newly diagnosed HIV-positive patients were included, with a mean age of 33±8.52, predominantly male. The majority of the patients belonged to the middle-aged group (76.9%). The demographic profile was similar to previous studies [9]. Most of our patients had no or low literacy levels (n=34; 52.30%), consistent with findings of earlier studies [10]. Marital status analysis revealed that 73.84% of patients were married, aligning with the survey by Bishnu et al. in 2014 (72.50%). The most common mode of transmission in our study was unprotected heterosexual contact (81.5%), in line with national figures and previous studies [9,11]. However, the transmission through blood and blood products was alarmingly high (7.7%), primarily attributed to blood transfusions performed in private hospitals using blood from private blood banks. This issue highlights the importance of stringent regulations and screening procedures [12]. Intravenous (IV) drug abuse, which was previously less common in our region [13], showed an increasing trend (4.6%). This behavior has played a critical role in the HIV epidemic in various regions, particularly Asia and southern Europe. Effective prevention strategies employed in developed countries, such as outreach to IV drug abusers, peer education programs, and social network interventions, are now being adopted in developing countries [12,14].

In the present study, the levels of IL-23 were considerably increased in persons who tested positive for HIV compared to a group of healthy individuals serving as controls (p < 0.01). IL-23 is crucial in the activation of the Th17 pathway. A heterodimeric receptor for IL-23 that transmits signals is made up of both a distinct IL-23 receptor (IL-23R) chain and the IL-12 receptor beta 1 (IL-12Rβ1) chain [15]. Furthermore, the study discovered a strong positive correlation between CD4+ T cell counts and IL-23 levels (r = 0.93, p < 0.01) among individuals with HIV, indicating that IL-23 may contribute to supporting CD4+ T cell populations in HIV-infected individuals. The elevated IL-23 levels could potentially help mitigate the adverse effects of HIV replication on the immune system. Notably, this research represents the first report of a positive relationship between IL-23 and CD4+ T cell counts in treatment-naive HIV seropositive individuals.

The study additionally examined IL-27 levels in HIV-infected individuals and observed a significant elevation in IL-27 levels compared to healthy controls (p < 0.001). This finding is consistent with previous reports describing the inhibition of HIV-1 replication by IL-27, where the increased cytokine levels are attributed to the early-stage immune response [16,17]. IL-27 is a member of the IL-6/IL-12 family produced by macrophages, endothelial cells, and dendritic cells. IL-27 stimulates Th1 responses and enhances the survival of naive T and B lymphocytes [7]. IL-27 inhibits HIV-1 replication through various mechanisms. Fakruddin et al. and Imamichi et al. reported that IL-27 up-regulates APOBEC3G, leading to the suppression of HIV-1 replication [18,7]. Greenwell et al. further corroborated these findings by showing that IL-27 induces IFN expression, which subsequently up-regulates APOBEC proteins, thereby inhibiting HIV-1 [19]. Additionally, Kwon et al. found that IL-27 induces the production of IL-10 in monocytes, contributing to the inhibition of HIV-1 [20]. Dai et al. demonstrated that IL-27 down-regulates spectrin β non-erythrocyte 1 (SPTBN1), a necessary host factor for HIV-1 infection [21].

We also observed a significant positive association (r = 0.92, < 0.01) between IL-27 and CD4+ T cell counts. Our findings suggest that IL-27 promotes the expansion of naive CD4+ T cells in vivo. He et al., in their study of 120 HIV-infected treatment-naive Chinese individuals, also found a positive correlation between IL-27 levels and CD4+ T cell counts [17].

The results of our study strongly indicate that both IL-23 and IL-27 may play a pivotal role in directly inhibiting HIV replication. Additionally, these cytokines may facilitate the rapid expansion of naive CD4+ T cells, potentially mitigating the adverse consequences of HIV replication. Conversely, individuals with low titers of IL-23 and IL-27 may face challenges in effectively controlling the virus, as these cytokines are integral to the immune response against HIV infection.

Limitations of the study

This study has a few limitations. We did not estimate HIV viral loads in our analysis. Additionally, the study was conducted at a single referral center, so the findings may not fully represent HIV infection characteristics on a national or regional scale. The control group was relatively small, but potential bias was mitigated by the large effect size. The control group was used primarily for comparison of CD4 count, IL-23, and IL-27 levels.

## Conclusions

The present study contributes valuable insights into the cytokine profile, namely, IL-23 and IL-27 in HIV seropositive ART-naive cases. In our research, for the first time, we showed that IL-23 and IL-27 levels are significantly elevated in HIV-infected individuals from India. Moreover, we established a positive correlation between IL-23, IL-27, and CD4+ T cell counts. This signifies that these cytokines do respond to HIV infection and could potentially play a crucial role in restraining HIV replication and slowing down the progression of the disease. These findings emphasize the ongoing importance of comprehensive research in the ever-evolving landscape of HIV healthcare. Further exploration through non-human primate models will inform the potential of these cytokines as therapeutic agents, setting the stage for rigorous pre-clinical assessments of their efficacy and potency.
